# Glutaredoxin-1 modulates the NF-κB signaling pathway to activate inducible nitric oxide synthase in experimental necrotizing enterocolitis

**DOI:** 10.1016/j.omtm.2024.101214

**Published:** 2024-02-19

**Authors:** Yunfei Zhang, Mei Yan, Yingying Xia, Yingbin Yue, Shuli Wang, Yuhui Hu, Genjian Lai, Quanjiang Wu, Qianyang Liu, Xin Ding, Chunbao Guo

**Affiliations:** 1 Pediatric Center of the First Affiliated Hospital of Xinjiang Medical University, Urumqi, Xinjiang 830054, P.R. China; 2 Department of Gastrointestinal Surgery, Renshou People’s Hospital, Meishan, Sichuan, P.R. China; 3 Department of Pediatric Surgery, Women and Children’s Hospital, Chongqing Medical University, Chongqing, P.R. China; 4 Ministry of Education Key Laboratory of Child Development and Disorders, Children’s Hospital of Chongqing Medical University, Chongqing, P.R. China; 5 Department of Pediatric Surgery, Chongqing Health Center for Women and Children, Chongqing, China; 6 Department of Psychiatry, Xinjin District Second People’s Hospital, Chengdu, Sichuan, P.R. China

**Keywords:** necrotizing enterocolitis, NF-κB, inducible nitric oxide synthase, glutaredoxin-1, S-glutathionylation, oxidative stress

## Abstract

Inducible nitric oxide synthase (iNOS), regulated by nuclear factor kappa B (NF-κB), is crucial for intestinal inflammation and barrier injury in the progression of necrotizing enterocolitis (NEC). The NF-κB pathway is inhibited by S-glutathionylation of inhibitory κB kinase β (IKKβ), which can be restored by glutaredoxin-1 (Grx1). Thus, we aim to explore the role of Grx1 in experimental NEC. Wild-type (WT) and Grx1-knockout (Grx1^−/−^) mice were treated with an NEC-inducing regimen. Primary intestinal epithelial cells (IECs) were subjected to LPS treatment. The production of iNOS, NO, and inflammation injuries were assessed. NF-κB and involved signaling pathways were also explored. The severity of NEC was attenuated in Grx1^−/−^ mice. Grx1 ablation promoted IKKβ glutathionylation, NF-κB inactivation, and decreased iNOS, NO, and O_2_^·–^ production in NEC mice. Furthermore, Grx1 ablation restrained proinflammatory cytokines and cell apoptosis, ameliorated intestinal barrier damage, and promoted proliferation in NEC mice. Grx1 ablation protected NEC through iNOS and NO inhibition, which related to S-glutathionylation of IKKβ to inhibit NF-κB signaling. Grx1-related signaling pathways provide a new therapeutic target for NEC.

## Graphical abstract

**Figure:**
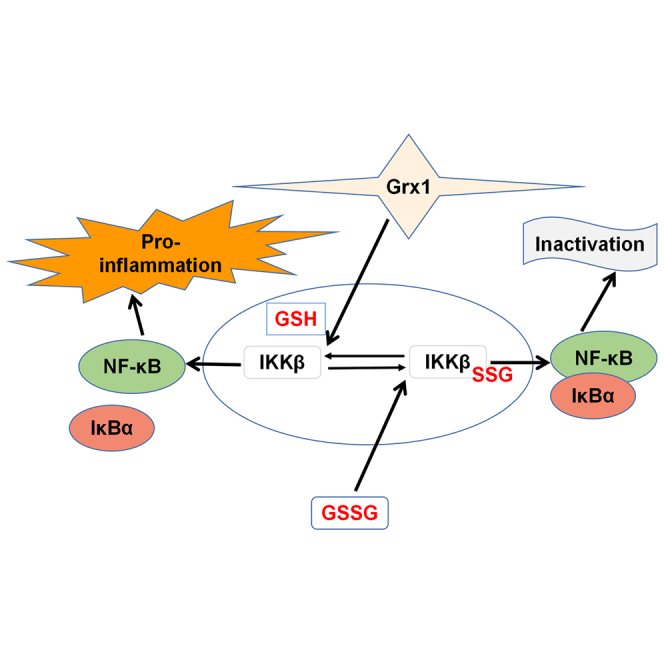

## Teaser

Zhang and colleagues explored the role of glutaredoxin-1 (Grx1) in necrotizing enterocolitis (NEC). Grx1 ablation protects NEC by reducing intestinal inflammation, cell apoptosis, and intestinal barrier damage. Mechanically, Grx1 deletion increases IKKβ S-glutathionylation and inhibits the NF-κB signaling pathway that reduces iNOS/NO production.

## Introduction

Necrotizing enterocolitis (NEC) is a common gastrointestinal emergency that is characterized by an increased inflammatory response and necrosis in premature intestines, correlating with high morbidity and mortality.^1^^,^^2^^,^^3^ Although NEC predominantly affects neonates, several risk factors have been identified, including prematurity, formula feeding, hypoxia, intestinal ischemia, and bacterial infection, and these have been linked to the etiology of NEC.^4^ Current studies indicate that intestinal injuries, for instance, microvascular disruption and hypoxiation, serve as the initial events that lead to bacterial translocation over the epithelial barrier, thereby amplifying innate immune system reactions.^5^^,^^6^^,^^7^ Certain luminal bacteria and their constituents promote the development of inflammatory factors, including proinflammatory cytokines, nitric oxide (NO), and peroxynitrite (ONOO^·–^).^8^^,^^9^^,^^10^^,^^11^ Although this scenario is widely accepted, the exact molecular mechanism involved in the pathogenesis of NEC remains undefined.

NO, produced by inducible nitric oxide synthase (iNOS), plays a pivotal role in intestinal barrier deterioration by inducing enterocyte death and inhibits epithelial restoration processes. ONOO^·–^, a powerful oxidant produced by the interaction of NO with superoxide (O_2_^·−^), is thought to mediate cytotoxic effects.^12^ However, the factors regulating iNOS overexpression in the gut are not fully understood, hampering efforts to create NO/iNOS-targeted therapeutics.

Nuclear factor kappa B (NF-κB) is a transcription factor involved in prosurvival, proinflammatory, and immunological regulatory sequences. NF-κB dysregulation has been associated with several persistent inflammatory conditions, including tumors, asthma, and sepsis.^13^^,^^14^ NF-κB is a heterodimer consisting of RelA and p50 subunits that is sequestered in an inactive form in the cell cytoplasm with inhibitory κB α (IκBα). Upon a specific IKB kinase β (IKKβ) activation, IκBα phosphorylation leads to polyubiquitination and proteasomal degradation of the inhibitory molecule. This process exposes a nuclear localization signal, translocating the NF-κB to the cell nucleus and transcriptional stimulation of downstream target genes, including the iNOS.^13^^,^^15^^,^^16^^,^^17^^,^^18^ Studies indicate that S-glutathionylation of IKKβ suppresses its kinase activity and prevents the degradation of IκBα and the DNA binding of RelA/p50 dimers. Therefore, it is a critical mechanism for controlling NF-κB activity.

Glutathione (GSH) is the major antioxidant in cells, which is converted to glutathione disulfide (GSSG) under oxidative stress. GSSG promoted proteins S-glutathionylated (PSSG) that can be reversed by Grx1. S-glutathionylation has arisen as a critical oxidative thiol modification that controls signaling molecules and transcription factors^19^^,^^20^ and affects the functionality of various proteins.^21^^,^^22^^,^^23^

Glutaredoxin-1 (Grx1) is a cytosolic enzyme that catalyzes protein deglutathionylation.^24^ Grx1 modulates inflammatory mediator synthesis by controlling S-glutathionylation-sensitive signaling pathways, such as NF-κB.^25^ Overexpression of Grx1 decreases IKKβ S-glutathionylation and enhances NF-κB activation, subsequently promoting the synthesis of inflammatory mediators.^26^ Conversely, the knockdown of Grx1 decreases the production of inflammatory mediators by controlling the S-glutathionylation-NF-κB signaling pathway.^25^ This correlation suggests the role of Grx1 in controlling the activation of iNOS through S-glutathionylation-NF-κB signaling in NEC pathogenesis.

Given the well-documented role of NF-κB in controlling inflammation and the relationship between Grx1 and S-glutathionylation, we aimed to investigate the role of Grx1 in NF-κB activation in the intestines of NEC mouse pups. Our findings underscore the pivotal role of Grx1 in modulating NF-κB activity and generating proinflammatory mediators, linking it to the iNOS/NO signaling pathway.

## Results

### Grx1 ablation decreases the production of iNOS and NO

**Figure 1:**
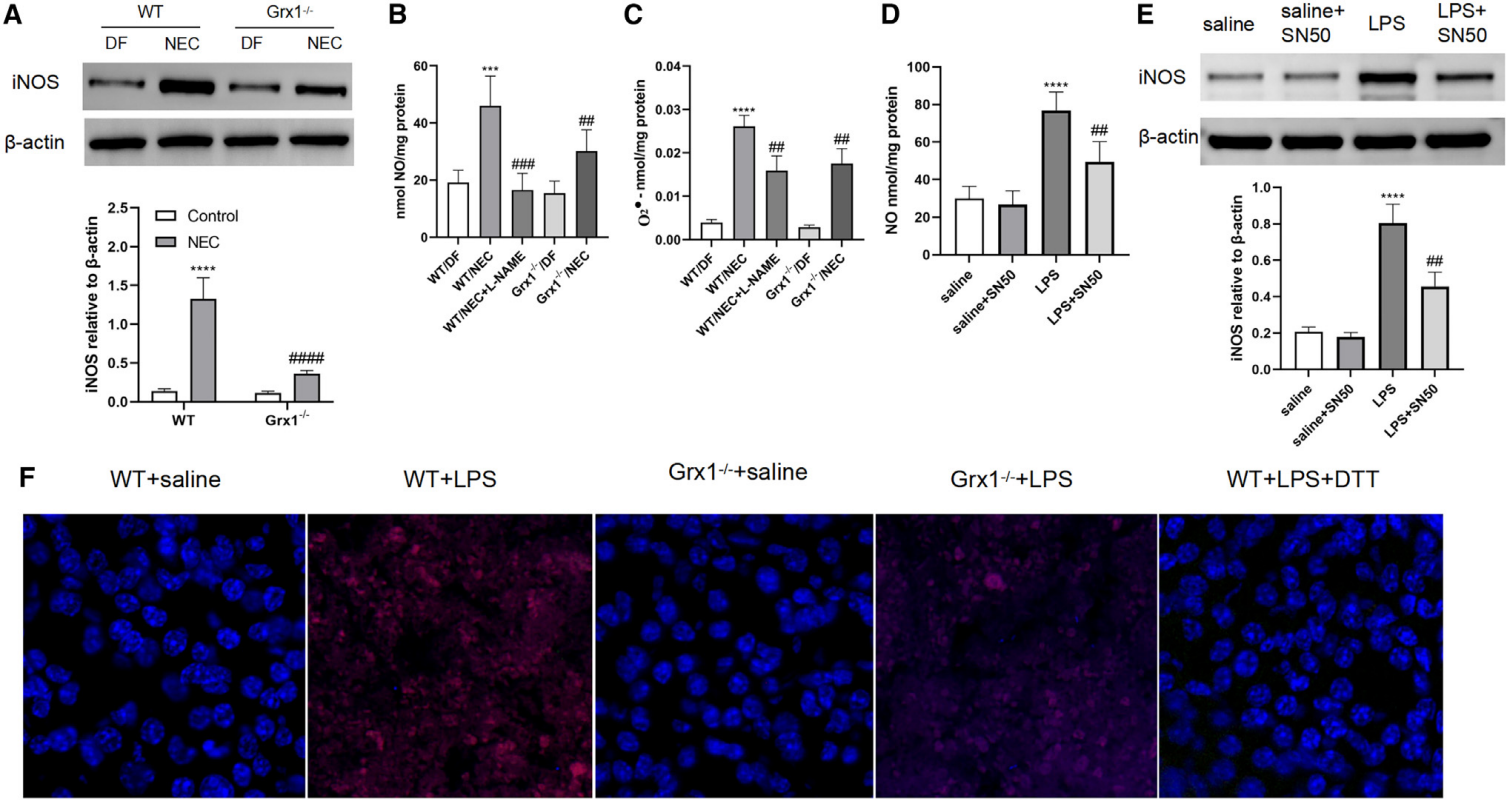
Evaluation of iNOS, NO and O_2_^·–^ (A) iNOS expression of intestines were analyzed by western blot. Bottom: densitometry analysis of iNOS normalized by β-actin. ∗∗∗∗p < 0.0001 vs. WT/dwarf (DF); ####p < 0.01 vs. WT/NEC. (B and C) NO (B) and O_2_^·–^ (C) production was evaluated in intestinal tissue between the groups. ∗∗∗p < 0.001, ∗∗∗∗p < 0.0001 vs. WT/DF mice; ###p < 0.001, ####p < 0.0001 vs. WT/NEC. (D) NO production was assessed in the IECs. (E) Western blot was performed for iNOS expression assessment on IECs, Bottom: densitometry analysis of iNOS normalized by β-actin. ∗∗∗∗p < 0.0001 vs. saline; ##p < 0.01 vs. LPS. (F) Representative fluorescence micrographs with DHE labeling for intracellular ROS detection. Two-sided 1-way ANOVA was used for data comparison with post hoc Tukey test (n = 6–8 mice per group; data are given as means ± SEMs).

iNOS is accountable for the high levels of NO that exert detrimental effects during inflammation. We explored the impact of Grx1 on iNOS expression in NEC mice. Endogenous iNOS protein levels were low or undetectable in Grx1^−/−^ and wild-type (WT) mice. NEC stress induced a marked increase in iNOS, and Grx1−/− significantly decreased the induction of iNOS (Figure 1A).

We then quantified NO production in NEC mice. An increase in NO and O_2_^·–^ production after NEC was detected in isolated intestines (Figures 1B and 1C). Grx1^−/−^ mice displayed reduced NO levels at baseline, and following NEC treatment, Grx1^−/−^ led to a significant reduction in NO production (Figure 1B). Furthermore, Grx1^−/−^ pups produced considerably less O_2_^·–^ than the WT after NEC stress (Figure 1C). The iNOS inhibitor NG-monomethyl-l-arginine, monoacetate salt suppressed NO signaling in NEC, implying that the source of NO is reliant on iNOS (Figure 1B). Therefore, Grx1^−/−^ was associated with the expression of iNOS, with a significant reduction in NO generation in NEC mice.

We then examined O_2_^·–^ generation in single-cell suspensions of intestinal epithelial cells (IECs) that are isolated from the same mice using confocal microscopy, identifying fluorescence from oxidized dihydroethidium (DHE). Exposure of IECs to lipopolysaccharide (LPS) (100 nmol/L, 1 h) improved the DHE-derived fluorescence signals significantly, which was attenuated by Grx1^−/−^ (Figure 1F). A reduction in DHE fluorescence by DTT in cells supported the switch from NO to O_2_^·–^ (Figure 1F).

To determine whether expression of iNOS and production of NO were mediated by NF-κB, we used the NF-κB activation inhibitor SN-50. In resting conditions, treating IECs with SN-50 reduced the production of NO and iNOS slightly. IECs showed a marked increase in iNOS and NO production after exposure to the LPS. However, the LPS-induced NO synthesis and iNOS activation were prevented by SN-50 significantly (Figures 1D and 1E), suggesting that NF-κB mediates the regulation of iNOS in IECs.

### Grx1 ablation reduces proinflammatory cytokines that associated with iNOS signaling

**Figure 2:**
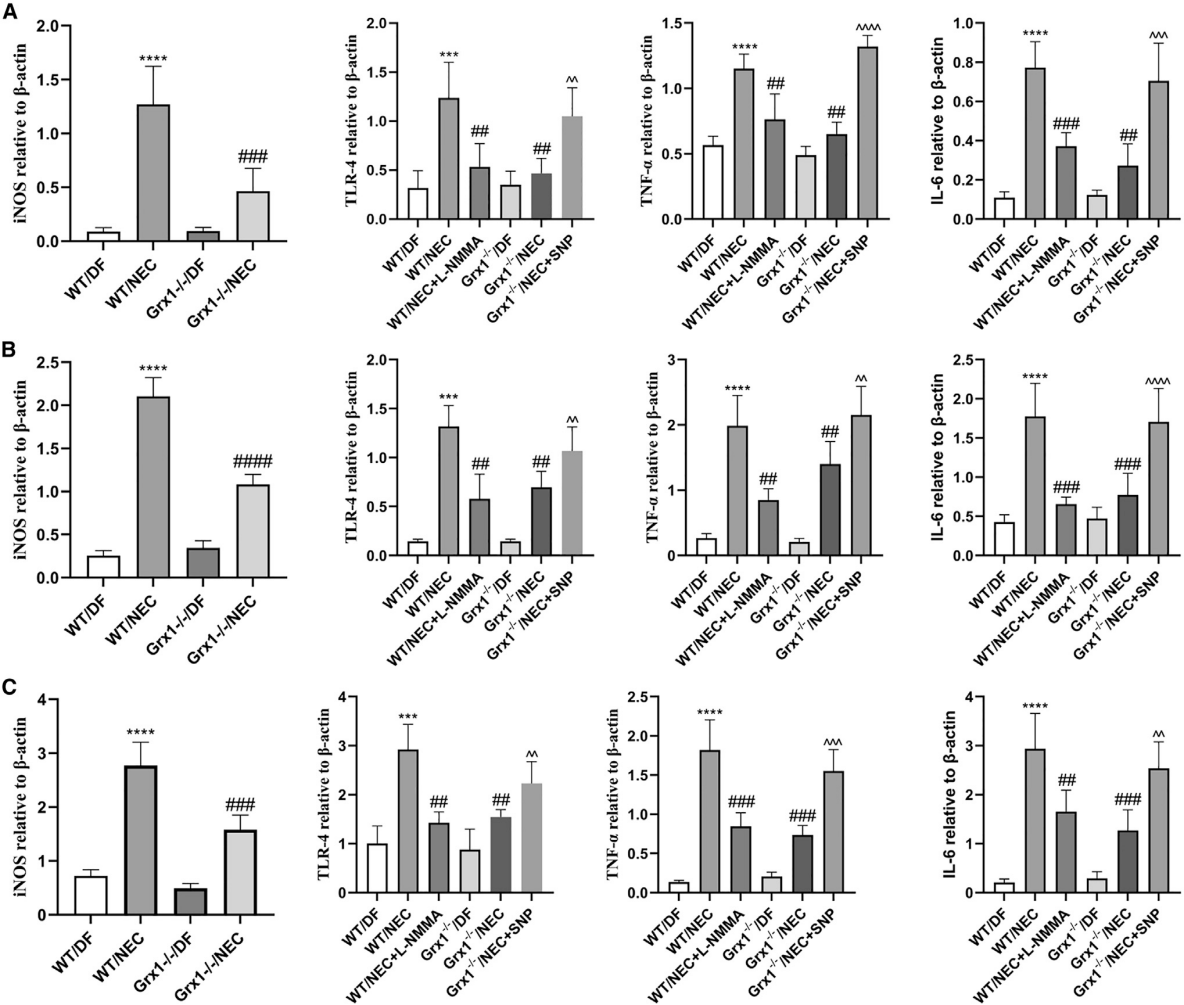
Proinflammatory cytokines evaluation in NEC mice (A–C) iNOS, TLR4, TNF-α, and IL-6 mRNA were detected in intestinal tissue (A), IECs (B), and peritoneal macrophages (C) by RT-PCR in different groups. Two-sided 1-way ANOVA was used for data comparison with post hoc Tukey test (n = 6–8 mice per group; data are presented as means ± SEMs). ∗∗∗p < 0.001, ∗∗∗∗p < 0.0001 vs. WT/DF mice; ##p < 0.01, ###p < 0.001 vs. WT/NEC; ˆˆp < 0.01,ˆˆˆp < 0.001, ˆˆˆˆp < 0.0001 vs. Grx1^−/−^/NEC.

We sought to determine whether Grx1-mediated iNOS could modulate proinflammatory cytokine activation in the pathophysiology of NEC, given that iNOS-derived NO contributes to intestinal inflammation. Toll-like receptor 4 (TLR4), tumor necrosis factor α (TNF-α), and the proinflammatory cytokine interleukin-6 (IL-6) are widely used to indicate inflammation. We first performed real-time PCR (RT-PCR) to evaluate the expression of iNOS, TLR4, TNF-α, and IL-6 on intestinal tissue. NEC stress significantly elevated the levels of iNOS, TLR4, TNF-α, and IL-6 mRNA in the intestinal tissue; however, the NO inhibitor *N*(ω)-nitro-l-arginine methylester (l-NAME) administration or Grx1^−/−^ reduced this expression (Figure 2A). Moreover, NO donor sodium nitroprusside (SNP) abrogated this anti-inflammatory effect of Grx1^−/−^ (Figure 2A).

We then isolated IECs and peritoneal macrophages from WT and Grx1^−/−^ mice. LPS exposure increased the expression of iNOS, TNF-α, TLR4, and IL-6 in both IECs and peritoneal macrophages from WT mice; however, this was attenuated by Grx1 ablation (Figures 2B and 2C). Moreover, SNP significantly promoted the expression of TNF-α, TLR4, and IL-6 in IECs and peritoneal macrophages, whereas l-NAME inhibited this, indicating the proinflammatory effects of NO (Figures 2B and 2C). Together, these data suggest that Grx1 functions as a proinflammatory mediator involved in iNOS signaling.

### Grx1 ablation promotes IEC migration and proliferation via NO signaling

**Figure 3:**
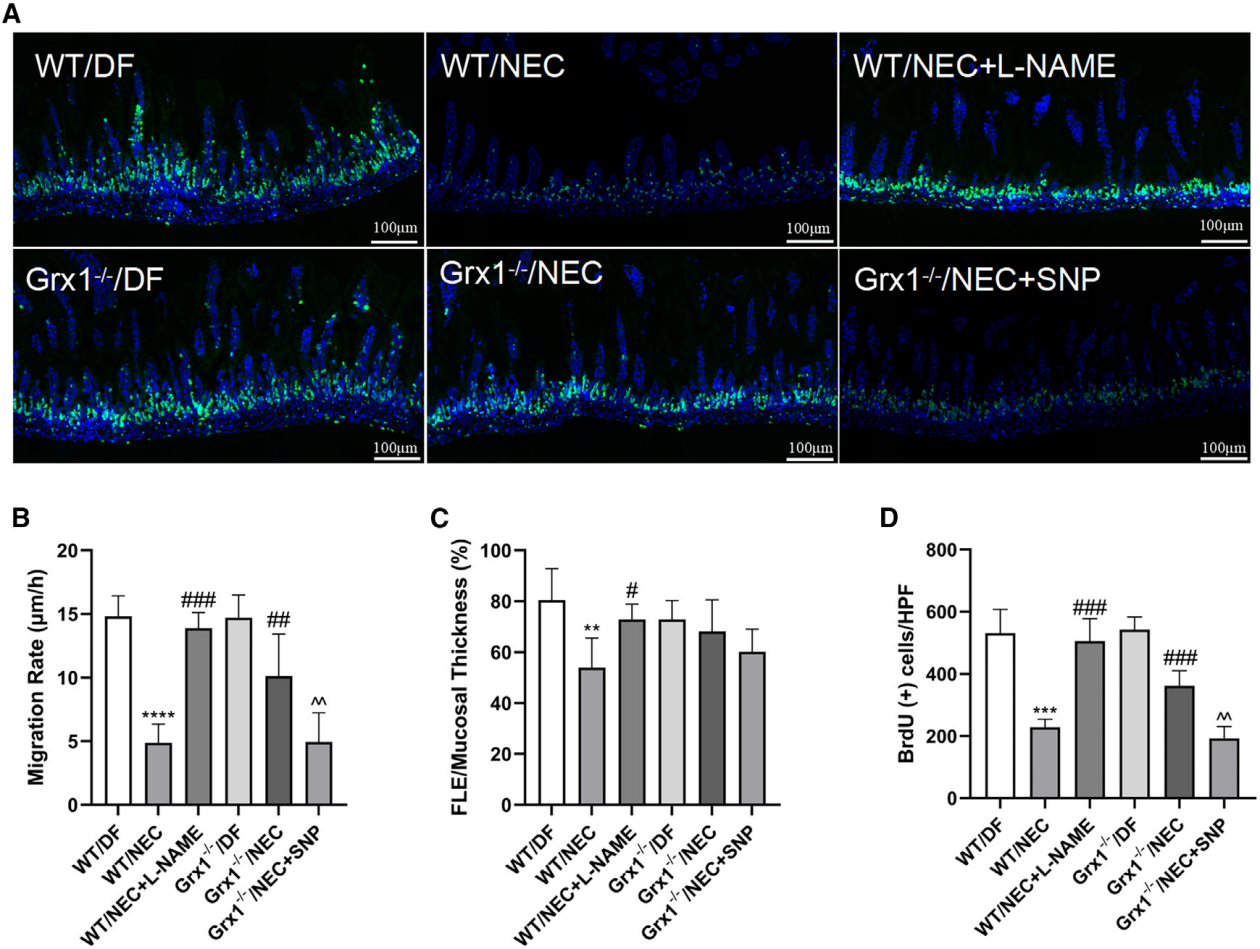
Grx1 regulates IEC proliferation and migration (A) Representative photomicrographs of BrdU immunostaining of the intestine. Scale bar: 100 μm. (B) IEC movement rate (measuring the distance from the bottom of the crypt to the foremost labeled enterocyte [FLE], with the migration rate [lm/h] calculated as FLE/18). (C) IEC movement (characterized as FLE/complete thickness of the mucosa × 100%). (D) IEC multiplication (characterized as BrdU^+^ cells/high-power field). n = 6 animals per group, 6 fields/animal. For data comparison, 2-sided 1-way ANOVA was used with post hoc Tukey test (n = 6–8 mice per group; data are presented as means ± SEMs). ∗∗p < 0.01, ∗∗∗p < 0.001, ∗∗∗∗p < 0.0001 vs. WT/DF mice; #p < 0.05, ##p < 0.01, ###p < 0.001 vs. WT/NEC; ˆˆp < 0.01 vs. Grx1^−/−^/NEC.

We then investigated whether Grx1 influences IEC proliferation and migration in the intestine, because both of these mechanisms are important in NEC pathogenesis. IECs were identified in intestinal tissue slices using bromodeoxyuridine (BrdU) immunostaining (Figure 3A). In the mouse model, NEC treatment severely impaired IEC migration. However, Grx1^−/−^ significantly improved this deficit (Figures 3B and 3C). BrdU^+^ cells were used to evaluate IEC proliferation. NEC stress significantly reduced IEC proliferation, which was attenuated by Grx1^−/−^ (Figure 3D). Moreover, inhibiting NO generation with l-NAME promoted IEC migration and proliferation in NEC mice; SNP administration abrogated the beneficial effects of Grx1^−/−^ on IEC functions (Figures 3B–3D). These findings suggest that Grx1 ablation contributes to IEC migration and proliferation, which is associated with iNOS/NO signaling in the development of NEC.

### Grx1 ablation ameliorates iNOS-mediated apoptosis and intestinal barrier damage

**Figure 4:**
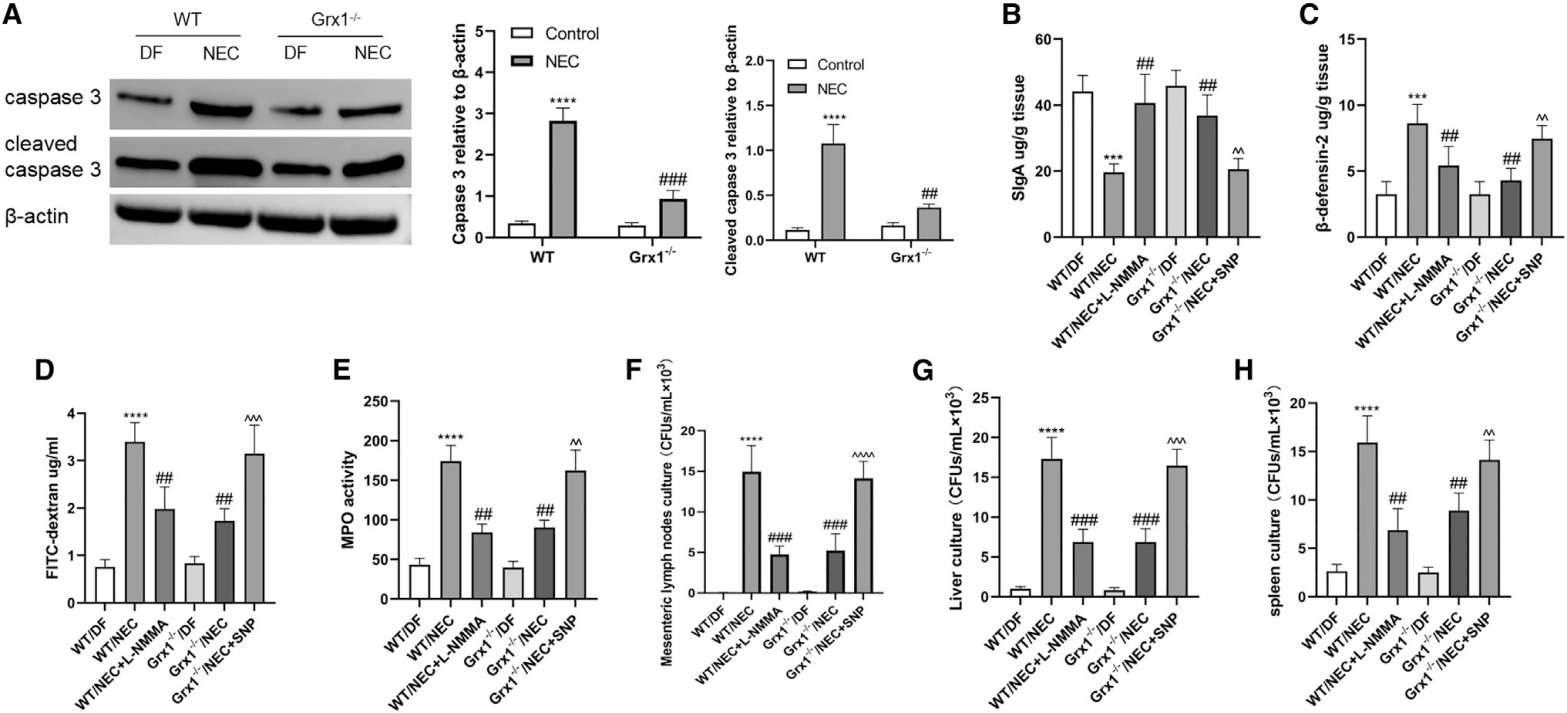
Apoptosis and intestinal barrier injury evaluation (A) Immunoblotting analysis of caspase-3 and cleaved caspase-3 proteins in intestine. Right: densitometry analysis of left images. (B) SIgA was estimated from the terminal ileum. (C) The centralization of β-defensin 2 was estimated in the distal ileum. (D) Serum FITC-dextran concentrations in groups were detected. (E) MPO activity was assessed in ileum. (F–H) Bacterial growth was quantified in mesenteric lymph nodes (F), liver (G), and spleen (H) of mice. The data represented 3 independent experiments. For data comparison, 2-sided 1-way ANOVA was used with post hoc Tukey test (n = 6–8 mice per group were adopted; data are presented as means ± SEMs). ∗∗∗p < 0.001, ∗∗∗∗p < 0.0001 vs. WT/DF mice; ##p < 0.01, ###p < 0.001 vs. WT/NEC; ˆˆp < 0.01,ˆˆˆp < 0.001, ˆˆˆˆp < 0.0001 vs. Grx1^−/−^/NEC.

We next investigated the role of Grx1 in influencing iNOS and its subsequent impact on intestinal cell apoptosis during NEC development. Grx1^−/−^ reduced intestinal apoptosis in NEC pups, as shown by SDS-PAGE analysis of caspase-3 and cleaved caspase-3 (Figure 4A). The mucosal barrier was disrupted during NEC stress, as evidenced by secretory immunoglobulin A (SIgA) elevation (Figure 4B), β-defensin-2 suppression (Figure 4C), as well as fluorescein isothiocyanate (FITC)-dextran exudation (Figure 4D); these detrimental effects were mitigated by Grx1^−/−^. In addition, the changes in myeloperoxidase (MPO) activity indicated neutrophil infiltration reduction (Figure 4E). We also investigated whether Grx1 affects the magnitude of bacterial translocation. NEC stress increased bacterial translocation over the intestinal barrier into the mesenteric lymph nodes (Figure 4F), liver (Figure 4G), and spleen (Figure 4H). This extent was ameliorated by Grx1^−/−^ or l-NAME administration. However, SNP abrogated this protective effect of Grx1^−/−^. These data suggest that Grx1 ablation ameliorates intestinal apoptosis and intestinal barrier damage in NEC through iNOS/NO signaling.

### Grx1 ablation impedes the development of NEC

**Figure 5:**
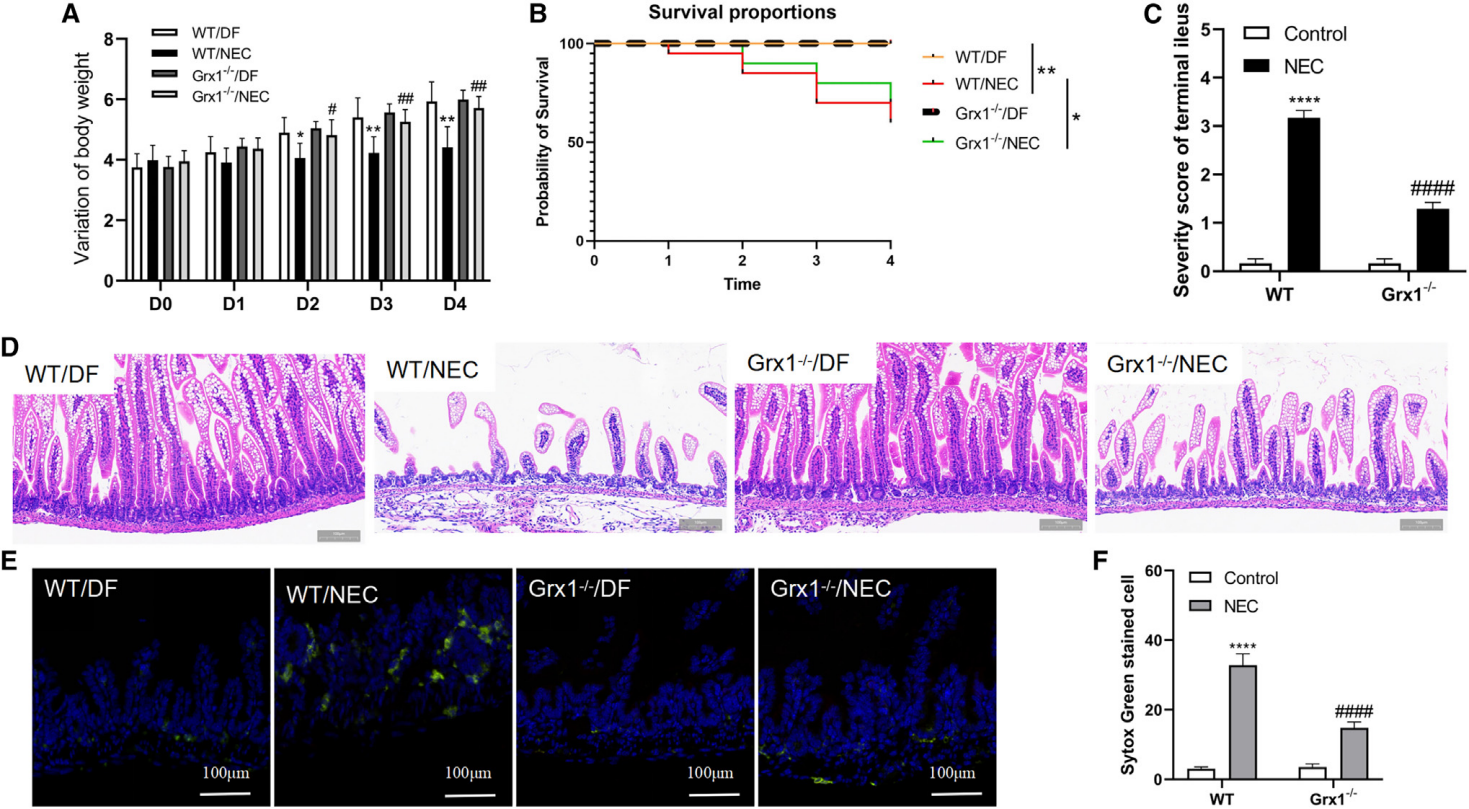
Grx1 deficiency decreases the severity of experimental NEC (A) Weight changes among different groups. (B) Survival curves in different groups. (C) Severity scores were computed based on morphological changes. (D) Morphology of the ileum was shown. (E) Represent SYTOX Green staining was shown between groups. (F) Groups were compared in terms of necrotic cells. A 2-sided 1-way ANOVA with a Tukey post hoc test (n = 10–15 mice per group; data are presented as means ± SEMs). ∗p < 0.05, ∗∗p < 0.01, ∗∗∗∗p < 0.0001 vs. WT/DF mice; #p < 0.05, ##p < 0.01, ####p < 0.0001 vs. WT/NEC mice.

To examine whether Grx1 influences the progression of NEC, we produced NEC in Grx1^−/−^ and WT mice. All of the groups’ body weights were measured. Although both NEC groups exhibited significant weight loss, the Grx1^−/−^/NEC group lost body weight more slowly than the WT/NEC group (Figure 5A). Mortality rates were also investigated. Survival curves showed that NEC treatment decreased postpartum survival rates significantly. However, Grx1^−/−^ slowed this progression (Figure 5B).

In terms of histological alterations, WT/NEC mice manifested more pronounced epithelial cell loss, complete villus necrosis, and transmural necrosis than Grx1^−/−^/NEC mice (Figure 5D). After NEC stress, the intestinal severity score in Grx1^−/−^ mice was considerably lower than that in WT mice (Figure 5C). Furthermore, necrosis in the intestinal epithelium, as evidenced by SYTOX Green staining, a necrosis marker that is used to detect cell death in the ileal epithelium based on its binding property to cellular nucleic acids present only in dead cells, was enhanced in NEC but considerably decreased by Grx1^−/−^ (Figures 5E and 5F). These results confirmed that Grx1 is critical in the development of NEC.

### Grx1 ablation increases S-glutathionylation of IKKβ, which inhibits NF-κB activation

**Figure 6:**
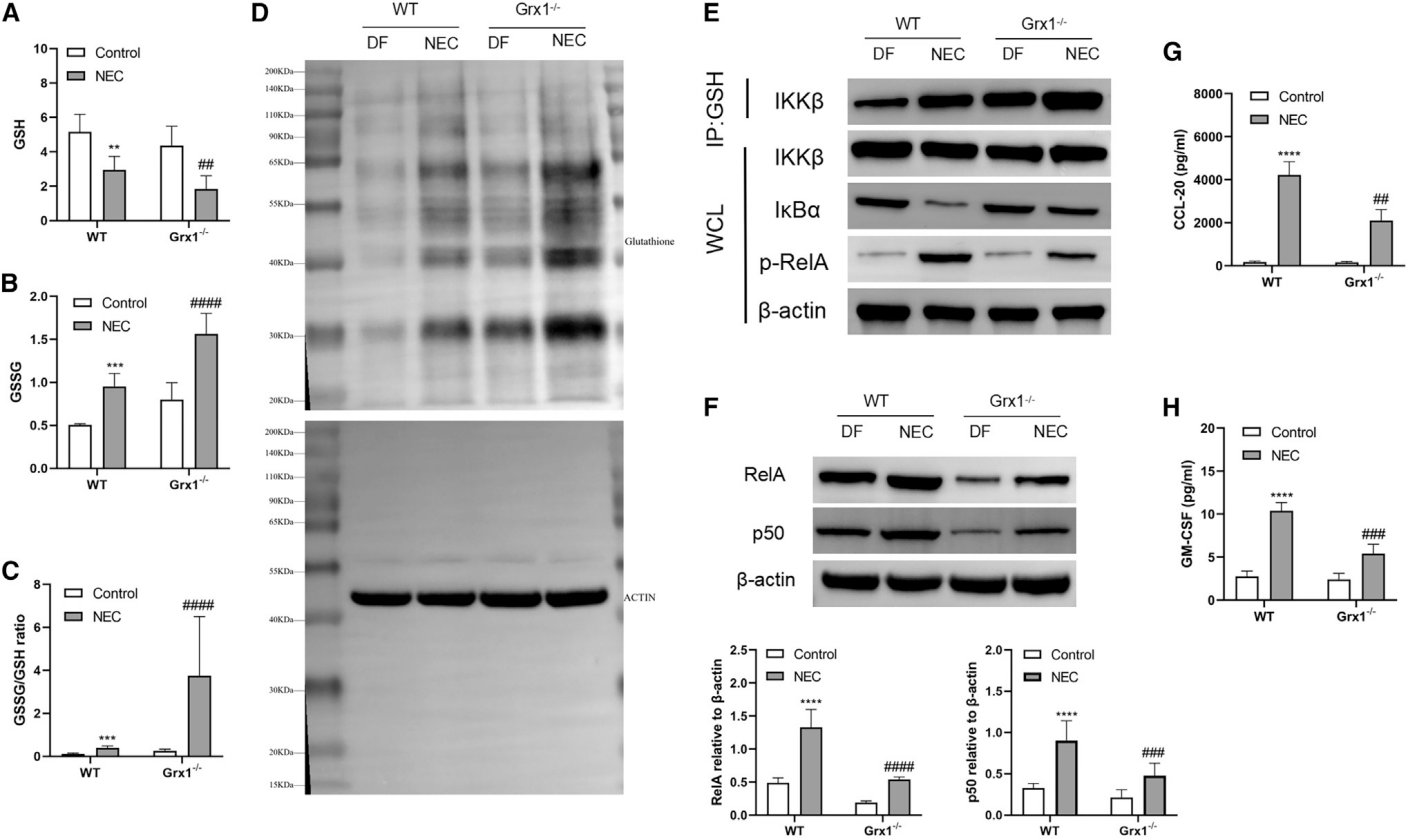
Assessment of NF-κB activation (A–C) Levels of GSH (A), GSSG (B), and the GSSG/GSH ratio (C) in the intestinal homogenates. (D) Western blot analysis of S-glutathionylation of intestinal tissue proteins in different groups. (E) Top: immunoprecipitation (IP) analysis of S-glutathionylation of IKKβ in intestinal tissue lysates; bottom: western blot analysis of whole-cell lysates (WCLs) for total IKKβ, IκBα, and phosphorylated RelA. (F) A western blot assay was used to analyze RelA and p50 proteins in intestines; bottom: densitometry analysis of RelA and p50 normalized by β-actin. (G and H) Evaluation of CCL-20 (G) and GM-CSF (H) concentrations in intestinal tissues using ELISA. For data comparison, we used a post hoc Tukey test with 2-sided 1-way ANOVA (n = 6–8 mice per group; data are presented as means ± SEMs). ∗∗∗∗p < 0.0001 vs. WT/DF mice; ##p < 0.01, ###p < 0.001 vs. WT/NEC mice. ∗p < 0.01, ∗∗p < 0.01, ####p < 0.001 vs. WT/NEC.

GSH is the major antioxidant that transformed into GSSG under oxidative stress. GSSG promoted proteins S-glutathionylated, which regulates the function of several proteins. Considering the function of Grx1 in controlling NF-κB and protein deglutathionylation catalysis, we investigated whether IKKβ S-glutathionylation (IKKβ-SSG) was reliant on Grx1. In our investigation, NEC stress decreased GSH levels significantly in mouse intestines. Conversely, the GSSG and GSSG/GSH ratio increased in experimental NEC intestines (Figures 6A, 6B, and 6C). Moreover, the GSSG and GSSG/GSH ratio increased more robustly in Grx1^−/−^ mice (Figures 6B and 6C). To investigate GSH-protein adducts in NEC mice, anti-GSH antibody was used for western blot assays on intestinal GSH proteins under nonreducing conditions. Under resting conditions, the intestinal GSH protein adducts increased slightly in Grx1^−/−^ mice as compared with WT mice. Although NEC treatment increased intestinal GSH levels in both types of mice, the increase was significantly higher in Grx1^−/−^ mice (Figure 6D). A slight increase in IKKβ-SSG was detected following NEC treatment; however, Grx1^−/−^ considerably intensified NEC-induced IKKβ-SSG (Figure 6E). This increases in IKKβ-SSG associated with an increase in IκBα and a decline in phosphor-RelA, suggesting sustained IKK inactivation (Figure 2E).

Assuming that S-glutathionylation of IKK limits its kinase activity, we explored potential function of Grx1 in controlling NF-κB activation and downstream cytokines. Intestinal RelA and p50 content noticeably increased following NEC treatment, whereas they decreased following Grx1^−/−^ (Figure 6F). In experimental NEC mice, the concentration of chemokine (C-C motif) ligand 20 (CCL-20) and granulocyte-macrophage colony-stimulating factor (GM-CSF), two cytokines regulated by NF-κB, also decreased following Grx1^−/−^ (Figures 6G and 6H). These outcomes revealed that Grx1^−/−^ leads to impaired activity of NF-κB and consequent decreases in downstream mediators following NEC treatment. Endogenous Grx1 is important for NEC-induced NF-κB activation.

## Discussion

To date, the potential role of Grx1 in intestinal damage during NEC remains underexplored. We investigated the role of Grx1 in controlling NF-κB activation, iNOS expression, S-glutathionylation, NO generation, and proinflammatory cytokine production in NEC mice. In an experimental animal model of NEC, we found that Grx1 ablation markedly mitigated the severity of NEC and increased the survival rate. Mechanically, Grx1^−/−^ increased the GSSG/GSH ratio due to the significantly increased GSSG and decreased GSH in NEC mice intestines, which provided a favorable circumstance for protein S-glutathionylation. Grx1^−/−^ increased IKKβ S-glutathionylation while it concurrently suppressed NF-κB activation and transcription. This led to diminished binding of NF-κB to the iNOS promoter, significantly restricting iNOS expression. Our findings also indicated that inhibiting iNOS by Grx1^−/−^ reduced NO production, correlating with a decline in proinflammatory cytokine levels. S-glutathionylation regulated by Grx1 plays a vital function in NEC pathogenesis.

NF-κB S-glutathionylation attenuates NO generation and iNOS expression in human neutrophils.^27^ These mechanisms may also be involved in NEC. NO is recognized as serving a paradoxical function in gut physiology. Endothelial NOS (eNOS) and iNOS are the major NO synthases that catalyze arginine to produce NO in the gut. eNOS is predominantly expressed in intestinal microcapillaries. eNOS is responsible for the low background NO that is essential for the preservation of mucosal capillaries and mucosal homeostasis.^5^^,^^28^ The high levels of NO are mainly induced by iNOS in the intestines. Enhanced iNOS expression has been seen in IECs from surgically removed NEC tissue samples.^29^ In an NEC rat model, a marked increase in both iNOS mRNA and protein expression leads to an increase in enterocyte apoptosis within the intestinal epithelium.^30^ Elevated NO concentrations during inflammation have a detrimental effect on the intestinal barrier via various mechanisms.^31^^,^^32^^,^^33^^,^^34^ In high concentrations, NO reacts with superoxide to form its reactive nitrogen derivative ONOO^·–^, which is highly toxic to epithelial cells.^12^ Both NO and ONOO^·–^ can disrupt intestinal barrier integrity by inducing apoptosis and necrosis in enterocytes.^35^ In addition, NO and its metabolites can hinder epithelial repair processes, including enterocyte proliferation and migration.^36^

In line with previous reports, we found that NEC stress increased iNOS and NO production, induced enterocyte apoptosis, attenuated enterocyte proliferation and migration, and increased gut barrier permeability, bacterial translocation, and proinflammatory cytokine production. In addition, Grx1 deletion reduced iNOS and NO production in NEC mice and attenuated NEC stress-induced adverse effects. Furthermore, a switch from NO to O_2_^·–^ generation in the intestines of pups with NEC was attenuated by Grx1^−/−^, verifying the important role of Grx1 in NO metabolism during oxidative stress through iNOS signaling.

Therefore, iNOS-derived NO is essential for intestinal homeostasis, and excessive NO leads to NEC epithelial injury. In addition, Grx1^−/−^ alleviates NEC stress-induced inflammatory injury, enhances IEC migration and proliferation, and fortifies the intestinal barrier function against bacterial invasion and mucosal permeability. Administration of the NO donor SNP abrogates the protective effect conferred by Grx1^−/−^, indicating that Grx1 regulates intestinal function through iNOS/NO signaling in NEC.

Extensive research has focused on the modulation of the iNOS gene in response to pathogen-associated molecular patterns in neutrophils and macrophages. This modulation is facilitated through the activation of the transcription factor NF-κB, which binds to the iNOS promoter, leading to the transcriptional activation of the iNOS gene.^16^^,^^37^

The S-glutathionylation of IKKβ inhibits the NF-κB pathway, which can be reversed by Grx1.^22^ In previous reports, Grx1 knockout cells exhibited increased S-glutathionylation of IKKβ. Conversely, upon the overexpression of Grx1, this S-glutathionylation of IKKβ was diminished, thereby activating the NF-κB.^25^ Grx1-overexpressing transgenic mice enhance endothelial NF-κB activity and inflammation^38^; therefore, Grx1 deletion potentially inactivates NF-κB through the glutathionylation of IKKβ.^22^^,^^23^

We found that Grx1^−/−^ improved the PSSG content in the intestines of NEC mice. The increased PSSG promoted IKKβ-SSG, which inhibited the phosphorylation and degradation of IκBα. This process diminished the translocation of RelA/p50 dimers to the nucleus, thereby preventing NF-κB activation and iNOS production. These results correspond with a 2010 study that reported that iNOS induction was suppressed in the aortic endothelium and hearts of Grx1^−/−^ mice.^39^ Furthermore, we found that the NF-κB activation inhibitor SN-50 significantly inhibited iNOS and NO production, attenuated the switch from NO to O_2_^·–^ generation, and reduced the production of reactive oxygen species (ROS) in NEC stress. These results indicated that iNOS expression is an NF-κB-dependent process. The observed reductions in iNOS and NO production can likely be attributed to the inactivation of the NF-κB pathway via the S-glutathionylation of IKKβ.

Previous studies have shown that cysteine 189 of IκBα can be S-glutathionylated, leading to decreased IKK phosphorylation and attenuated ubiquitination *in vitro*.^40^ This results in the reduced degradation of IKK and subsequent inhibition of NF-κB activation.^41^ Future research should focus on determining the potential interactions between Grx1 and IκBα, particularly regarding the S-glutathionylation of IκBα, and whether these processes affect IKK signalosome activation and/or assembly in NEC pathogenesis. Grx1 is ubiquitously expressed, and it remains unclear which types of occupant cells are important in the Grx1^−/−^ mice. More research is needed to determine the conditions under which Grx1 ablation is applied across distinct target cell types in NEC pathogenesis.

In conclusion, Grx1 ablation inhibited iNOS/NO production, protecting against NEC through GSH adducts on the NF-κB signaling pathway. Consequently, Grx1 downregulation could serve as a promising therapeutic target to mitigate NEC-associated intestinal damage.

## Materials and methods

### Animals

For each experimental protocol in the investigation, we obtained approval from Chongqing Medical University’s Institutional Animal Care and Use Committee (IACUC). Prof. Jingyu Li generously provided Grx1^−/−^ mice (C57BL/6J genetic background, Sichuan University, Chengdu, China). The Research Animal Center of Chongqing Medical University provided WT mice (C57BL/6J, Chongqing, China). Although the controls were allowed to breastfeed with their mothers, the investigational offspring (various genders) going through NEC induction were isolated from their mothers on postnatal day 5 (P5). The isolated pups (body weight: 3–4 g) were kept in 37°C incubators on a 12:12-h dark:light cycle and were offered bedding. NEC was induced in Grx1^−/−^ mice and WT mice between P5 and P9 using oral administration of LPS (4 mg/kg), hypoxia, and gavage feeding with hyperosmolar formula, as previously mentioned.^42^ On P9, the pups were euthanized, and the terminal ileum was collected for examination.

### GSH and GSSG determination

Levels of GSSG and reduced GSH were spectrophotometrically determined using a GSSG and GSH measurement kit (S0053, Beyotime, Shanghai, China).

### Morphological and histological assessment

Formalin-fixed terminal ileal specimens were sectioned at 4-μm intervals, stained with H&E, and inspected under a microscope. We scored intestinal damage severity, applying a standard histological scoring system via two researchers blinded to the remedy cohorts.^43^ Intestinal injury was graded on a 5-point scale: grade 0: no injury; grade 1: injury to villus tips or colonic epithelium, or mild separation of lamina propria; grade 2: mid-villus disruption and/or moderate separation of lamina propria; grade 3: complete villus disruption and/or severe separation and/or edema in submucosa; and grade 4: transmural injury. NEC is defined as mice with a grade 2 or above score.

### Western blot analysis

Snap-frozen terminal ileum tissues were homogenized and centrifuged and the supernatant was collected. The protein content was calculated by using the bicinchoninic acid approach. Equal amounts of proteins (40 mg) were subjected to SDS-PAGE, transferred to polyvinylidene difluoride membranes (IPFL00010, Millipore, Burlington, MA), and probed with primary antibodies. Membranes were blocked using QuickBlock Western (Beyotime) and subsequently incubated overnight at 4°C with primary antibodies, including β-actin (20536-1-AP, Proteintech, Rosemont, IL), cleaved caspase-3 (Santa Cruz Biotechnology, Dallas, TX), IκBα (Cell Signaling Technology, Danvers, MA), Grx1 (ab45953, Abcam, Cambridge, UK), IKKβ (ab124957, Abcam), RelA (ab32536, Abcam), *p*-RelA (ab76302, Abcam), and iNOS (ab178945, Abcam). Following primary incubation, membranes were treated with appropriate horseradish peroxidase-conjugated secondary antibodies for 1 h at room temperature to visualize immunoreactivity bands. The optical density of these bands was analyzed using ImageJ software (NIH, Bethesda, MD), and band intensities were captured with the Kodak Scientific Imaging System (Kodak, Rochester, NY).

### Detection of protein S-glutathionylation

The immunoprecipitation assay was conducted as described previously.^44^ To summarize, the protein was extracted using an SDS lysis buffer comprising 20 mM *N*-ethyl maleimide (Sigma-Aldrich, St. Louis, MO). We incubated 250 mg of protein with 1 mg/mL anti-PSSG antibody (Virogen, Watertown, MA) and recombinant protein G agarose beads, followed by western blot analysis. Before immunoprecipitation, we incubated the selected reagent control specimens, administering 50 mM DTT for 30 min, as previously stated.^45^

### NO and O_2_^·−^ anion assessment

We performed O_2_^·–^ and NO analyses using the total NO assay kit (Beyotime) as previously described.^46^ Intestinal sections were homogenized in sterile PBS (1 mL), and supernatants were collected postcentrifugation. For the O_2_^·–^ assay, the supernatants were incubated with a luminescent O_2_^·–^ test reagent, lucigenin (Sigma-Aldrich). Luminescence was detected using a luminometer (Turner Biosystems, Sunnyvale, CA) and expressed as relative light units/mg protein. Moreover, NEC pups were administered the NO synthase suppressor l-NAME before gavage feeding.^47^

### Intestinal permeability

According to the guideline for intestinal permeability,^46^ we administered FITC-conjugated dextran (70 kDa, Sigma-Aldrich) (40 mg/100 g body weight) intragastrically for 4 h. Then, we sacrificed the pups and obtained the blood for serum fluorescence determination.

### IEC and peritoneal macrophage isolation and culture

The protocol for the isolation and culture of primary IECs from mouse small intestines has been established.^48^ Specifically, the resected small intestine was washed twice in sterile medium, cultivated in EDTA solution (1.25% trypsin, 0.5 mmol/L) at normal temperature, and incubated in intestinal crypts with media that contained 200 U/mL collagenase type IV. Following dissociation, the IECs were washed and incubated with 5% CO_2_ at 32°C. These essential enterocyte cultures were affirmed by immunological strategy, as described previously.^49^ Peritoneal macrophages were gathered from mice through peritoneal lavage with 10 mL of super-cold PBS per animal. Cells were then turned down, resuspended in DMEM/F-12 medium (Life Advances, Carlsbad, CA), and adhered by plating on glass coverslips.^45^ We treated the isolated IECs and peritoneal macrophages with NF-κB activation inhibitor SN-50 (25 mM, Calbiochem, San Diego, CA) for 30 min and then treated them further with saline or LPS (1 mg/mL).^27^

### DHE staining for O_2_^·–^ detection

Staining was performed under the O_2_^·–^ detection regime, as described previously.^46^ Specifically, we incubated IECs with the O_2_^·–^-sensitive DHE dye (red color) (5 μM; Thermo Fisher Scientific, Waltham, MA) at 37°C for 30 min. We used DAPI (blue) to stain the nuclei (Sigma-Aldrich). We visualized and recorded ROS production (O_2_^·–^, 535 nm) with a confocal microscope (Leica TCS SP5). Quantitative analysis of the confocal images was performed using ImageJ software.

### Migration and proliferation of IEC tissue

To evaluate IEC proliferation and migration, subjects received intraperitoneal injections of BrdU. Eighteen hours postinjection, the subjects were euthanized, and immunofluorescence staining was performed on the terminal ileum in accordance with the established quantification protocol.^50^

### ELISA

GM-CSF and CCL-20 in homogenized intestinal samples were detected using the Duoset ELISA kits in accordance with the manufacturer’s instructions (R&D Systems, Minneapolis, MN).

### RT-PCR

Total RNA was extracted from mouse intestine segments using an RNA assay kit following the manufacturer’s guidelines (AG, Shanghai, China). The RNA quantity was evaluated. The RNA was converted to cDNA by a reverse transcription reagent kit (AG, Shanghai, China). The SYBR Premix Ex Taq (AG, Shanghai, China) on a CFX96 Touch Real-Time PCR Detection System (Bio-Rad, Hercules, CA) was used for amplification. Relative mRNA expression was calculated and normalized using the ΔΔCt method, referencing the β-actin gene expression. The sequences of the selected primers are presented in Table 1 (AG, Shanghai, China).

**Table 1:** Oligonucleotide sequences of primers used in this study

Gene	Forward sequence	Reverse sequence
β-actin	CATCCGTAAAGACCTCTATGCCAAC	ATGGAGCCACCGATCCACA
iNOS	TGACCATCATGGACCACCAC	ACCAGCCAAATCCAGTCTGC
TLR4	TTTATTCAGAGCCGTTGGTG	CAGAGGATTGTCCTCCCATT
TNF-α	CATCTTCTCAAAATTCGAGTGACAA	TGGGAGTAGACAAGGTACAACCC
IL-6	GGCTAAGGACCAAGACCATCCAA	TCTGACCACAGTGAGGAATGTCCA

### Statistical assessment

GraphPad Prism software version 4 was used for data processing. The outcomes are expressed as mean ± SEM, considering the normal distribution of data. For comparison, a one-way ANOVA with post-hoc Tukey analysis was employed. The log rank test was used to evaluate survival curves. p < 0.05 was regarded as statistically significant.

## Data and code availability

The datasets used and/or analyzed during the present study are available from the corresponding author on reasonable request.

## References

[1] Mara M.A., Good M., Weitkamp J.H. (2018). Innate and adaptive immunity in necrotizing enterocolitis [J]. Semin. Fetal Neonatal Med..

[2] Niño D.F., Sodhi C.P., Hackam D.J. (2016). Necrotizing enterocolitis: new insights into pathogenesis and mechanisms [J]. Nat. Rev. Gastroenterol. Hepatol..

[3] Neu J., Walker W.A. (2011). Necrotizing enterocolitis [J]. N. Engl. J. Med..

[4] Ford H.R., Sorrells D.L., Knisely A.S. (1996). Inflammatory cytokines, nitric oxide, and necrotizing enterocolitis [J]. Semin. Pediatr. Surg..

[5] Yazji I., Sodhi C.P., Lee E.K., Good M., Egan C.E., Afrazi A., Neal M.D., Jia H., Lin J., Ma C. (2013). Endothelial TLR4 activation impairs intestinal microcirculatory perfusion in necrotizing enterocolitis via eNOS-NO-nitrite signaling [J]. Proc. Natl. Acad. Sci. USA.

[6] Thänert R., Keen E.C., Dantas G., Warner B.B., Tarr P.I. (2021). Necrotizing Enterocolitis and the Microbiome: Current Status and Future Directions [J]. J. Infect. Dis..

[7] Lu P., Sodhi C.P., Jia H., Shaffiey S., Good M., Branca M.F., Hackam D.J. (2014). Animal models of gastrointestinal and liver diseases. Animal models of necrotizing enterocolitis: pathophysiology, translational relevance, and challenges [J]. Am. J. Physiol. Gastrointest. Liver Physiol..

[8] Chokshi N.K., Guner Y.S., Hunter C.J., Upperman J.S., Grishin A., Ford H.R. (2008). The role of nitric oxide in intestinal epithelial injury and restitution in neonatal necrotizing enterocolitis [J]. Semin. Perinatol..

[9] Managlia E., Yan X., De Plaen I.G. (2022). Intestinal Epithelial Barrier Function and Necrotizing Enterocolitis [J]. Newborn (Clarksville).

[10] Hackam D.J., Afrazi A., Good M., Sodhi C.P. (2013). Innate immune signaling in the pathogenesis of necrotizing enterocolitis [J]. Clin. Dev. Immunol..

[11] Sorrells D.L., Friend C., Koltuksuz U., Courcoulas A., Boyle P., Garrett M., Watkins S., Rowe M.I., Ford H.R. (1996). Inhibition of nitric oxide with aminoguanidine reduces bacterial translocation after endotoxin challenge in vivo [J]. Arch. Surg..

[12] FORD H.R. (2006). Mechanism of nitric oxide-mediated intestinal barrier failure: insight into the pathogenesis of necrotizing enterocolitis [J]. J. Pediatr. Surg..

[13] Yu H., Lin L., Zhang Z., Zhang H., Hu H. (2020). Targeting NF-κB pathway for the therapy of diseases: mechanism and clinical study [J]. Signal Transduct. Target. Ther..

[14] Kunnumakkara A.B., Shabnam B., Girisa S., Harsha C., Banik K., Devi T.B., Choudhury R., Sahu H., Parama D., Sailo B.L. (2020). Inflammation, NF-κB, and Chronic Diseases: How are They Linked? [J]. Crit. Rev. Immunol..

[15] Cinelli M.A., Do H.T., Miley G.P., Silverman R.B. (2020). Inducible nitric oxide synthase: Regulation, structure, and inhibition [J]. Med. Res. Rev..

[16] Sze S.C.W., Zhang L., Zhang S., Lin K., Ng T.B., Ng M.L., Lee K.F., Lam J.K.W., Zhang Z., Yung K.K.L. (2022). Aberrant Transferrin and Ferritin Upregulation Elicits Iron Accumulation and Oxidative Inflammaging Causing Ferroptosis and Undermines Estradiol Biosynthesis in Aging Rat Ovaries by Upregulating NF-Κb-Activated Inducible Nitric Oxide Synthase: First Demonstration of an Intricate Mechanism [J]. Int. J. Mol. Sci..

[17] Capece D., Verzella D., Flati I., Arboretto P., Cornice J., Franzoso G. (2022). NF-κB: blending metabolism, immunity, and inflammation [J]. Trends Immunol..

[18] Zhang G.Z., Liu M.Q., Chen H.W., Wu Z.L., Gao Y.C., Ma Z.J., He X.G., Kang X.W. (2021). NF-κB signalling pathways in nucleus pulposus cell function and intervertebral disc degeneration [J]. Cell Prolif..

[19] Wang Y., Yang J., Yi J. (2012). Redox sensing by proteins: oxidative modifications on cysteines and the consequent events [J]. Antioxid. Redox Signal..

[20] Vrettou S., Wirth B. (2022). S-Glutathionylation and S-Nitrosylation in Mitochondria: Focus on Homeostasis and Neurodegenerative Diseases [J]. Int. J. Mol. Sci..

[21] Matsui R., Ferran B., Oh A., Croteau D., Shao D., Han J., Pimentel D.R., Bachschmid M.M. (2020). Redox Regulation via Glutaredoxin-1 and Protein S-Glutathionylation [J]. Antioxid. Redox Signal..

[22] Reynaert N.L., van der Vliet A., Guala A.S., McGovern T., Hristova M., Pantano C., Heintz N.H., Heim J., Ho Y.S., Matthews D.E. (2006). Dynamic redox control of NF-kappaB through glutaredoxin-regulated S-glutathionylation of inhibitory kappaB kinase beta [J]. Proc. Natl. Acad. Sci. USA.

[23] Pineda-Molina E., Klatt P., Vázquez J., Marina A., García de Lacoba M., Pérez-Sala D., Lamas S. (2001). Glutathionylation of the p50 subunit of NF-kappaB: a mechanism for redox-induced inhibition of DNA binding [J]. Biochemistry.

[24] Watanabe Y., Murdoch C.E., Sano S., Ido Y., Bachschmid M.M., Cohen R.A., Matsui R. (2016). Glutathione adducts induced by ischemia and deletion of glutaredoxin-1 stabilize HIF-1α and improve limb revascularization [J]. Proc. Natl. Acad. Sci. USA.

[25] Aesif S.W., Anathy V., Kuipers I., Guala A.S., Reiss J.N., Ho Y.S., Janssen-Heininger Y.M.W. (2011). Ablation of glutaredoxin-1 attenuates lipopolysaccharide-induced lung inflammation and alveolar macrophage activation [J]. Am. J. Respir. Cell Mol. Biol..

[26] Aesif S.W., Kuipers I., van der Velden J., Tully J.E., Guala A.S., Anathy V., Sheely J.I., Reynaert N.L., Wouters E.F.M., van der Vliet A., Janssen-Heininger Y.M.W. (2011). Activation of the glutaredoxin-1 gene by nuclear factor κB enhances signaling [J]. Free Radic. Biol. Med..

[27] Singh A.K., Awasthi D., Dubey M., Nagarkoti S., Kumar A., Chandra T., Barthwal M.K., Tripathi A.K., Dikshit M. (2016). High oxidative stress adversely affects NFκB mediated induction of inducible nitric oxide synthase in human neutrophils: Implications in chronic myeloid leukemia [J]. Nitric Oxide.

[28] Jensen A.R., Drucker N.A., Khaneki S., Ferkowicz M.J., Markel T.A. (2017). Hydrogen sulfide improves intestinal recovery following ischemia by endothelial nitric oxide-dependent mechanisms [J]. Am. J. Physiol. Gastrointest. Liver Physiol..

[29] Ford H., Watkins S., Reblock K., Rowe M. (1997). The role of inflammatory cytokines and nitric oxide in the pathogenesis of necrotizing enterocolitis [J]. J. Pediatr. Surg..

[30] Jing Y., Peng F., Shan Y., Jiang J. (2018). Berberine reduces the occurrence of neonatal necrotizing enterocolitis by reducing the inflammatory response [J]. Exp. Ther. Med..

[31] Du Plessis J., Vanheel H., Janssen C.E.I., Roos L., Slavik T., Stivaktas P.I., Nieuwoudt M., van Wyk S.G., Vieira W., Pretorius E. (2013). Activated intestinal macrophages in patients with cirrhosis release NO and IL-6 that may disrupt intestinal barrier function [J]. J. Hepatol..

[32] Baumann A., Rajcic D., Brandt A., Sánchez V., Jung F., Staltner R., Nier A., Trauner M., Staufer K., Bergheim I. (2022). Alterations of nitric oxide homeostasis as trigger of intestinal barrier dysfunction in non-alcoholic fatty liver disease [J]. J. Cell Mol. Med..

[33] Mu K., Yu S., Kitts D.D. (2019). The Role of Nitric Oxide in Regulating Intestinal Redox Status and Intestinal Epithelial Cell Functionality [J]. Int. J. Mol. Sci..

[34] Wang H.C., Chou H.C., Chen C.M. (2023). Molecular Mechanisms of Hyperoxia-Induced Neonatal Intestinal Injury [J]. Int. J. Mol. Sci..

[35] Buonpane C., Yuan C., Wood D., Ares G., Klonoski S.C., Hunter C.J. (2020). ROCK1 inhibitor stabilizes E-cadherin and improves barrier function in experimental necrotizing enterocolitis [J]. Am. J. Physiol. Gastrointest. Liver Physiol..

[36] Grishin A., Bowling J., Bell B., Wang J., Ford H.R. (2016). Roles of nitric oxide and intestinal microbiota in the pathogenesis of necrotizing enterocolitis [J]. J. Pediatr. Surg..

[37] Potoka D.A., Upperman J.S., Zhang X.R., Kaplan J.R., Corey S.J., Grishin A., Zamora R., Ford H.R. (2003). Peroxynitrite inhibits enterocyte proliferation and modulates Src kinase activity in vitro [J]. Am. J. Physiol. Gastrointest. Liver Physiol..

[38] Murdoch C.E., Shuler M., Haeussler D.J.F., Kikuchi R., Bearelly P., Han J., Watanabe Y., Fuster J.J., Walsh K., Ho Y.S. (2014). Glutaredoxin-1 up-regulation induces soluble vascular endothelial growth factor receptor 1, attenuating post-ischemia limb revascularization [J]. J. Biol. Chem..

[39] Bachschmid M.M., Xu S., Maitland-Toolan K.A., Ho Y.S., Cohen R.A., Matsui R. (2010). Attenuated cardiovascular hypertrophy and oxidant generation in response to angiotensin II infusion in glutaredoxin-1 knockout mice [J]. Free Radic. Biol. Med..

[40] Kil I.S., Kim S.Y., Park J.W. (2008). Glutathionylation regulates IkappaB [J]. Biochem. Biophys. Res. Commun..

[41] Seidel P., Roth M., Ge Q., Merfort I., S'Ng C.T., Ammit A.J. (2011). IκBα glutathionylation and reduced histone H3 phosphorylation inhibit eotaxin and RANTES [J]. Eur. Respir. J..

[42] Zani A., Zani-Ruttenstock E., Peyvandi F., Lee C., Li B., Pierro A. (2016). A spectrum of intestinal injury models in neonatal mice [J]. Pediatr. Surg. Int..

[43] Ran-Ressler R.R., Khailova L., Arganbright K.M., Adkins-Rieck C.K., Jouni Z.E., Koren O., Ley R.E., Brenna J.T., Dvorak B. (2011). Branched chain fatty acids reduce the incidence of necrotizing enterocolitis and alter gastrointestinal microbial ecology in a neonatal rat model [J]. PLoS One.

[44] Li X., Li X., Shang Q., Gao Z., Hao F., Guo H., Guo C. (2017). Fecal microbiota transplantation (FMT) could reverse the severity of experimental necrotizing enterocolitis (NEC) via oxidative stress modulation [J]. Free Radic. Biol. Med..

[45] Anathy V., Roberson E., Cunniff B., Nolin J.D., Hoffman S., Spiess P., Guala A.S., Lahue K.G., Goldman D., Flemer S. (2012). Oxidative processing of latent Fas in the endoplasmic reticulum controls the strength of apoptosis [J]. Mol. Cell Biol..

[46] Shang Q., Bao L., Guo H., Hao F., Luo Q., Chen J., Guo C. (2017). Contribution of glutaredoxin-1 to S-glutathionylation of endothelial nitric oxide synthase for mesenteric nitric oxide generation in experimental necrotizing enterocolitis [J]. Transl. Res..

[47] Chan K.L., Hui C.W.C., Chan K.W., Fung P.C.W., Wo J.Y.H., Tipoe G., Tam P.K.H. (2002). Revisiting ischemia and reperfusion injury as a possible cause of necrotizing enterocolitis: Role of nitric oxide and superoxide dismutase [J]. J. Pediatr. Surg..

[48] Nanthakumar N., Meng D., Goldstein A.M., Zhu W., Lu L., Uauy R., Llanos A., Claud E.C., Walker W.A. (2011). The mechanism of excessive intestinal inflammation in necrotizing enterocolitis: an immature innate immune response [J]. PLoS One.

[49] Lu L., Khan A., Walker W.A. (2009). ADP-ribosylation factors regulate the development of CT signaling in immature human enterocytes [J]. Am. J. Physiol. Gastrointest. Liver Physiol..

[50] Feng J., Besner G.E. (2007). Heparin-binding epidermal growth factor-like growth factor promotes enterocyte migration and proliferation in neonatal rats with necrotizing enterocolitis [J]. J. Pediatr. Surg..

